# Clinicopathological and prognostic insights into Embryonal Tumors with Multilayered Rosettes (ETMRs)

**DOI:** 10.1186/s13000-026-01766-y

**Published:** 2026-02-10

**Authors:** Noura A. A. Ebrahim, Heba A. Abdelbaky, Ahmed Mustafa Abd Elsalam, Mustafa A Hussein, Nancy H Amin

**Affiliations:** 1 Oncologic Pathology Department, National Cancer Institute (NCI) - Cairo University, Cairo, Egypt; 2 Pediatric Oncology Department, National Cancer Institute (NCI)-Cairo University, Cairo, Egypt

**Keywords:** ETMR, Pediatric brain tumors, C19MC amplification, LIN28A, Histopathology, Molecular diagnostics, Prognosis, Rosette formation, Mitotic index, Necrosis, Chemoradiotherapy

## Abstract

**Background:**

Embryonal tumors with multilayered rosettes (ETMRs) are rare, highly aggressive pediatric brain neoplasms characterized by early onset and dismal prognosis. This study presents a comprehensive clinicopathological and molecular analysis of ETMR cases diagnosed over a 14-year period at National Cancer Institute, Cairo University, with a focus on diagnostic features, clinical presentation, and survival outcomes.

**Methods:**

A retrospective review of 35 patients with histopathologically confirmed ETMRs was conducted. Demographic data, clinical symptoms, neuroimaging findings, histopathologic features-including rosette formation, mitotic activity, necrosis, and Ki67 proliferation index-as well as molecular analyses for C19MC amplification and LIN28A expression were evaluated. Kaplan-Meier survival curves and univariable Cox regression were used to assess prognostic associations.

**Results:**

The cohort comprised 17 females and 18 males, with a median age of 36 months. Common presenting symptoms included signs of raised intracranial pressure, seizures, and motor deficits. Gross total resection was achieved in 43% of patients, and 48% received adjuvant chemoradiotherapy.Histopathologic examination consistently revealed ependymoblastic rosettes (true multilayered rosettes) and high mitotic activity. LIN28A was diffusely expressed in all assessable cases. Molecular confirmation by PCR testing was done in 20 cases. The median overall survival was 19 months. Factors associated with inferior survival included incomplete surgical resection, absence of adjuvant therapy, and presence of necrosis and high mitotic index.

**Conclusion:**

ETMRs demonstrate consistent histological and immunohistochemical features that can guide diagnosis in resource-limited settings. Despite therapeutic advances, prognosis remains poor, underscoring the urgent need for novel therapeutic strategies. Molecular testing for C19MC amplification and LIN28A expression supports diagnostic confirmation and may hold future prognostic or therapeutic relevance.

## Introduction

 Embryonal tumors with multilayered rosettes (ETMR) represent a highly aggressive category of central nervous system neoplasm classified as WHO grade 4, first recognized as a distinct tumor type based on characteristic histopathological features approximately twenty years ago [1–4]. Before their current classification, these malignancies were encompassed within the diagnostic category of central nervous system primitive neuroectodermal tumors (CNS-PNET) [5, 6]. A pivotal discovery identified amplification of the C19MC miRNA cluster at chromosome 19q13.42 as the molecular hallmark of ETMR, establishing it as a defining genetic alteration [7]. ETMRs exhibit significant histological and anatomical diversity, encompassing multiple histologic patterns including embryonal tumors with abundant neuropil and true rosettes (ETANTR), ependymoblastoma (EBL), and medulloepithelioma (MEPL) [8, 9]. Aunifying molecular feature across these histologic subtypes amplifies the C19MC locus, with LIN28A expression as a reliable immunohistochemical surrogate [9, 10]. Recent studies have identified DICER1 mutations in tumors lacking C19MC amplification, expanding the molecular spectrum of ETMR [11, 12].

Extent of surgical resection correlates with improved survival outcomes when feasible in localized disease, akin to other pediatric CNS malignancies. Current therapeutic approaches typically employ protocols designed for high-risk medulloblastoma. However, the rarity of ETMR and its recent oncologic delineation from other CNS embryonal tumors has precluded the development of prospectively validated treatment regimens incorporating chemotherapy or radiotherapy [13–15]. This study presents a decade-long institutional experience with ETMR, analyzing clinicopathological characteristics, therapeutic strategies, and survival outcomes to enhance understanding of this rare neoplasm.

## Materials and methods

### Ethical considerations

The study protocol was reviewed and approved by the Institutional Review Board (IRB) of the National Cancer Institute, Cairo University (Approval Number: PA2502-501-095-197). All procedures were performed in alignment with the ethical standards of the institutional research committee and the principles outlined in the Declaration of Helsinki. As this was a retrospective analysis utilizing de-identified patient data, the requirement for informed consent was formally waived by the IRB. Strict measures were taken to preserve patient confidentiality, ensuring that no personal identifiers were accessed, recorded, or disclosed during data collection, analysis, or dissemination of results.

### Case selection

This retrospective study included patients diagnosed with ETMR between January 2009 and December 2023 at the Department of Oncologic Pathology, National Cancer Institute, Cairo University. Cases were identified through the institutional pathology archives using diagnostic terms corresponding to ETMR, including the previous nomenclature of embryonal tumors with abundant neuropil and true rosettes (ETANTR), ependymoblastoma, and medulloepithelioma, in line with evolving WHO classifications. Only those cases confirmed on histology and reviewed by neuropathologists were included.

### Histopathological evaluation and immunohistochemistry

Archived formalin-fixed, paraffin-embedded (FFPE) tissue blocks were retrieved for all included cases, and corresponding hematoxylin and eosin (H&E)-stained slides were re-evaluated by four oncologic pathologists with expertise in pediatric neuro-oncology. Diagnosis was confirmed based on morphological criteria consistent with ETMR, as defined by the 2021 WHO Classification of CNS Tumors.

The 2021 WHO Classification of CNS Tumors’ morphological criteria for ETMR were used to confirm the diagnosis. The evaluation encompassed several histopathological parameters, including nuclear pleomorphism, cytologic atypia, mitotic activity, the density of multilayered rosettes, and the presence of neuropil-like matrix elements. Special attention was given to the identification of multilayered rosettes—particularly ependymoblastic rosettes—which served as a key diagnostic hallmark. Histologically, the tumors often exhibited highly cellular areas composed of primitive neuroepithelial cells arranged in sheets, ribbons, or perivascular pseudorosettes, frequently set within a neuropil-like background. Mitotic activity was measured by counting mitotic figures in 10 consecutive high-power fields (HPFs) within the most proliferative regions, and necrosis was documented as either focal or diffuse, with its extent expressed as a percentage of tumor tissue. This structured assessment protocol provided a reliable basis for consistent grading of tumor differentiation across the entire cohort.

Immunohistochemical analysis was performed on 4 μm-thick formalin-fixed, paraffin-embedded (FFPE) tissue sections using the Ventana BenchMark XT automated staining system (Ventana Medical Systems, Tucson, AZ, USA). The following primary antibodies were applied: anti-LIN28A (clone EPR4640, Ventana), a recognized marker for embryonal stemness and a definitive diagnostic marker for ETMR; anti-synaptophysin (clone SP11, Ventana) to assess neuronal differentiation; anti-cytokeratin (AE1/AE3, Ventana) for epithelial differentiation; anti-Nestin (clone 10C2, Ventana) as a neural progenitor cell marker; anti-S100 (clone 4C4.9, Ventana) to assess glial and neural differentiation; and anti-Ki-67 (clone 30 − 9, Ventana) to evaluate cellular proliferative activity. Heat-induced epitope retrieval was performed using the Ventana Cell Conditioning 1 (CC1) solution, followed by visualization with an ultraView Universal DAB Detection Kit (Ventana). Appropriate positive control tissues and negative reagent controls were included in each staining batch to validate assay performance.

LIN28A expression was interpreted as positive when strong, diffuse cytoplasmic staining was observed in tumor cells. Synaptophysin staining was assessed for cytoplasmic positivity in neuropil and tumor cells. CK expression was evaluated for cytoplasmic and/or membranous positivity, particularly in epithelial-like areas. Nestin positivity was scored based on the extent and intensity of cytoplasmic staining.The Ki-67 proliferative index was assessed in areas of maximal nuclear labeling and was recorded as a percentage of positively stained tumor nuclei.

The integration of histomorphological findings with the immunohistochemical profile, particularly strong LIN28A positivity in conjunction with multilayered rosettes, was considered sufficient for a diagnosis of ETMR, however further molecular confirmation was carried out.

### Molecular analysis

A total of 35 cases diagnosed as ETMR were initially considered for molecular analysis, with adequate tumor content (> 70%). Of these, seven cases were excluded due to suboptimal DNA integrity or inadequate amplification on preliminary assessment. DNA extraction was subsequently performed on the remaining 28 cases using 10-µm thick FFPE tissue sections and the QIAamp DNA FFPE Tissue Kit (Qiagen, Germany), following the manufacturer’s protocol. After evaluating DNA yield and purity, eight additional samples were excluded owing to insufficient concentration or poor quality, leaving 20 cases eligible for quantitative PCR (qPCR) analysis. Genomic DNA was extracted from 10-µm thick FFPE tissue sections using the QIAamp DNA FFPE Tissue Kit, and samples were assessed for DNA concentration and purity. For C19MC amplification analysis, quantitative real-time PCR targeting miR-517a, miR-519d, and miR-520 g was performed, with relative quantification determined by the 2^−ΔΔCT^method. Additionally, for C19MC-negative cases, DICER gene mutations were analyzed through targeted sequencing of DICER1 exonic regions using the Illumina MiSeq platform, with bioinformatics analysis identifying mutations associated with tumorigenesis [9, 16–20].

### Statistical analysis

All statistical analyses were conducted using both IBM SPSS Statistics (version 28.0; IBM Corp., Armonk, NY) and R software (version 4.3.2; R Foundation for Statistical Computing, Vienna, Austria) to ensure cross-validation of results and optimize analysis robustness. Descriptive statistics were utilized to summarize demographic, clinical, and pathological characteristics, with categorical variables expressed as frequencies and percentages, while continuous variables were reported as medians and interquartile ranges (IQR) due to the non-parametric distribution of most data. For survival analysis, overall survival (OS) was defined as the interval from initial diagnosis to death from any cause or last follow-up, and progression-free survival (PFS) was measured from the date of surgical intervention to documented disease progression, recurrence, or death, whichever occurred first. Patients remaining alive without progression were censored at last follow-up. Kaplan-Meier survival curves were generated for both OS and PFS estimates, and comparisons between survival distributions of categorical subgroups-such as extent of resection, adjuvant therapy, and histological parameters-were performed using the log-rank test. Median survival times and their corresponding 95% confidence intervals (CI) were reported. Cox proportional hazards regression models were applied to investigate potential associations between survival estimates and clinicopathological variables, including age, sex, tumor site, surgical extent, adjuvant treatment, mitotic index, necrosis, and Ki-67 labeling index, with hazard ratios (HR), 95% CIs, and p-values calculated; significance was set at *p* < 0.05. To assess associations between categorical variables, cross-tabulation analyses were initially performed using Pearson’s Chi-square test. However, considering the limited cohort size (*n* = 35) and the occurrence of low expected cell counts, Monte Carlo simulation (10,000 iterations) was employed to derive more reliable p-values that adjust for small-sample variability. Additionally, Cramér’s V coefficient was computed to measure the effect size of associations between categorical variables, offering a dimensionless index ranging from 0 to 1, irrespective of sample size. All hypothesis tests were two-tailed, with statistical significance defined as *p* < 0.05.

## Results

### Patient demographics and clinical features

This study included 35 pediatric patients with histologically confirmed ETMRs. The mean age at diagnosis was 3.11 ± 1.41 years (range: 1–6 years), and there was an almost equal sex distribution (51.4% male). Family history of cancer was positive in only 5.7% (*n* = 2).

The median time from symptom onset to diagnosis was 40 days. Headache (88.6%), vomiting (65.7%), and nausea (54.3%) were the most common presenting symptoms, while motor weakness and convulsions were reported in 48.6% and 22.9% of cases, respectively. Altered consciousness was seen in 17.1%. The demographic and clinical profiles of the studied cohort are summarized in Table 1.

**Table 1:** Demographic and clinical characteristics

Variable	Value / Distribution
Age at diagnosis (years)	Mean = 3.11 ± 1.41; Median = 3
Sex	Male: 18 (51.4%), Female: 17 (48.6%)
Family history of cancer	Yes: 2 (5.7%), No: 33 (94.3%)
Time to diagnosis (days)	Median = 40 (IQR: 30–60)
Headache	88.6% (*n* = 31)
Vomiting	65.7% (*n* = 23)
Nausea	54.3% (*n* = 19)
Motor weakness	48.6% (*n* = 17)
Convulsions	22.9% (*n* = 8)
Altered consciousness	17.1% (*n* = 6)

### Tumor characteristics and histopathology

Tumors were primarily supratentorial (74.3%) with a similar distribution between left (45.7%) and right hemispheres (42.9%). Tumors were most frequently located in the left frontoparietal region (6 cases, 17.1%), followed by the left parietal lobe (5 cases, 14.3%), and both the right frontoparietal region and right parietal lobe (4 cases each, 11.4%). Other supratentorial sites included the left parieto-occipital region (3 cases, 8.6%), left temporo-parietal region (2 cases, 5.7%), right occipito-parietal region (2 cases, 5.7%), left frontal lobe (2 cases, 5.7%), and right temporo-parietal region (1 case, 2.9%). Infratentorial involvement was observed in the right cerebellar hemisphere (2 cases, 5.7%), left cerebellar hemisphere (1 case, 2.9%), and brainstem (2 cases, 5.7%). Ventricular involvement was identified in one case each of the midline 4th ventricle and 3rd ventricle (2 cases, 5.7%). The sellar and suprasellar region was affected in 2 cases (5.7%).

Tumor size was ≥ 5 cm in 48.6% of patients. The mean tumor size was 6.53 ± 1.54 cm. Gross total resection (GTR) was achieved in 43% of cases.

Histopathological subtypes included ETANTR (37.1%), medulloepithelioma (34.3%), and ependymoblastoma (28.6%). Most tumors showed moderate differentiation (42.9%), with high proliferative indices (mean Ki-67 = 50.7%) and mitotic activity (mean mitoses = 31.9/10 HPF). Necrosis was present in nearly all tumors (mean necrotic area = 15.8%), and the median number of rosettes per HPF was 3.

LIN28A, a hallmark marker of ETMR, demonstrated uniform strong cytoplasmic positivity across all analyzed cases (*n* = 35; 100%).

INI-1 expression was retained in all examined cases (*n* = 35). GFAP showed positivity in 4 cases, while synaptophysin was expressed in 21 cases. Nestin was consistently positive across all cases (*n* = 35; 100%), with staining patterns ranging from focal to diffuse. Cytokeratin (CK) demonstrated focal to patchy positivity in 21 cases, and S100 protein was positive in 10 cases.

The Ki-67 proliferation index ranged from 25% to 90%, with a median around 40%–50%. Over 65% of tumors showed Ki-67 ≥ 50%, indicating high proliferative activity, consistent with the aggressive biological behavior of ETMR.Detailed pathological and surgical characteristics of the tumors are presented in Table 2.

**Table 2:** Tumor pathological and surgical characteristics

Characteristic	Number (percentage)
Location	Supratentorial: 26 (74.3%)
Laterality	Left: 16, Right: 15, Midline: 4
Tumor size (cm)	Mean = 6.53 ± 1.54
Histologic subtypes	ETANTR (37.1%), Medulloepithelioma (34.3%), Ependymoblastoma (28.6%)
Degree of differentiation	Moderate (42.9%), Poor (20%), Well (37.1%)
Ki-67 index (%)	Mean = 50.7 ± 21.9
Mitoses/10 HPF	Mean = 31.9 ± 11.3
Necrosis (%)	Mean = 15.8 ± 15.0
Rosettes/HPF	Median = 3 (range 0–5)
Type of surgery	Partial Resection (57%), Gross Total Resection (43%)
Metastasis at diagnosis	14.30%

**Fig. 1:**
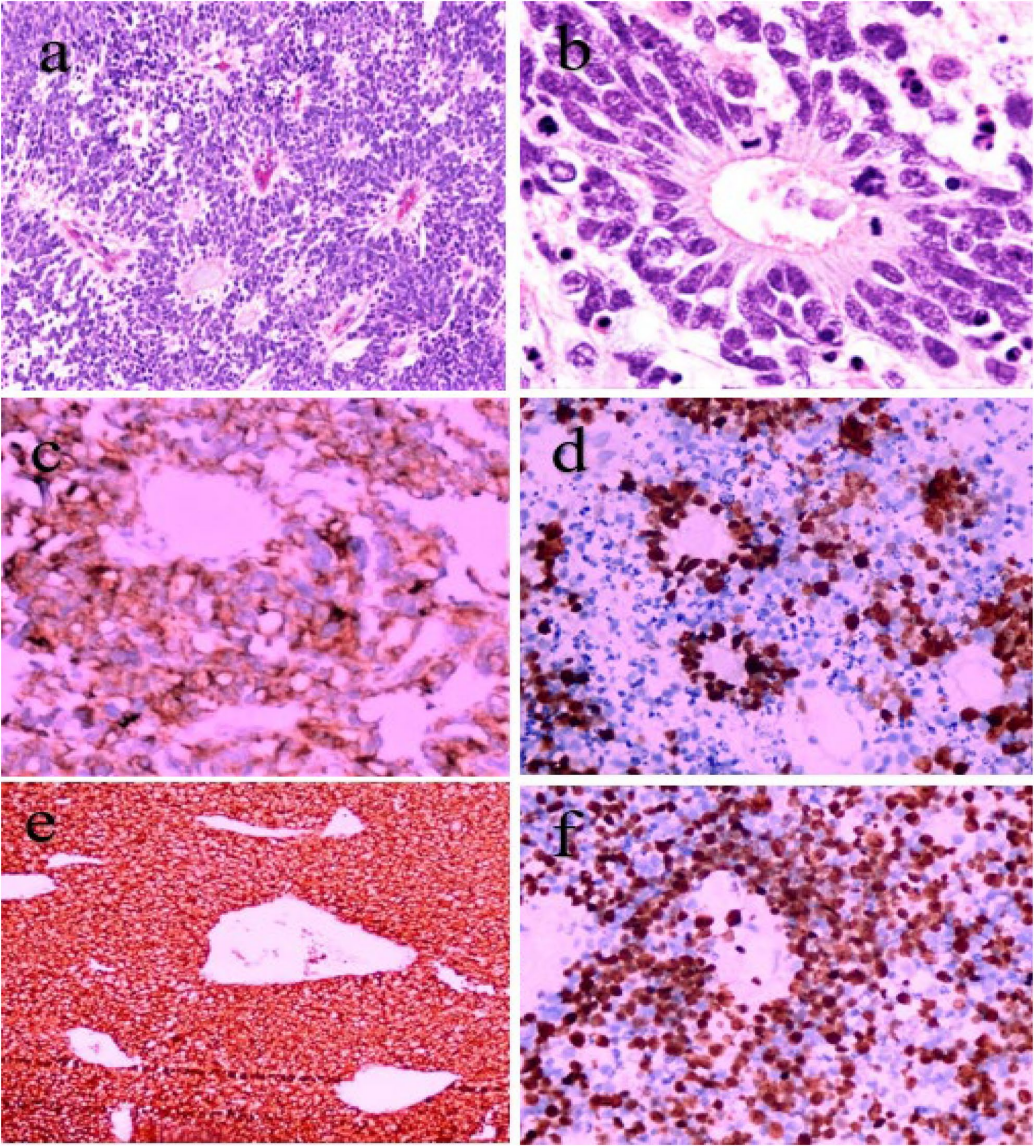
Histopathological and immunohistochemical features of an ependymoblastoma case. **a** Hematoxylin and eosin (H&E) stained section at 200× magnification displaying numerous true multilayered rosettes, characteristic of ependymoblastoma. **b** H&E-stained section at 400× magnification highlighting moderate nuclear atypia and frequent mitotic figures, indicating active tumor proliferation. **c** Immunohistochemistry for LIN28A reveals moderate to strong cytoplasmic positivity in most of the neuroepithelial tumor cells, supporting a diagnosis within the ETMR spectrum. **d** S100 shows nuclear immunoreactivity, particularly accentuated in the rosette-forming regions. **e** Nestin staining demonstrates diffuse cytoplasmic positivity, consistent with a primitive neuroectodermal phenotype. **f** Ki-67 labeling index is estimated at 60–70%, reflecting a high proliferative activity typical of this aggressive tumor type

**Fig. 2:**
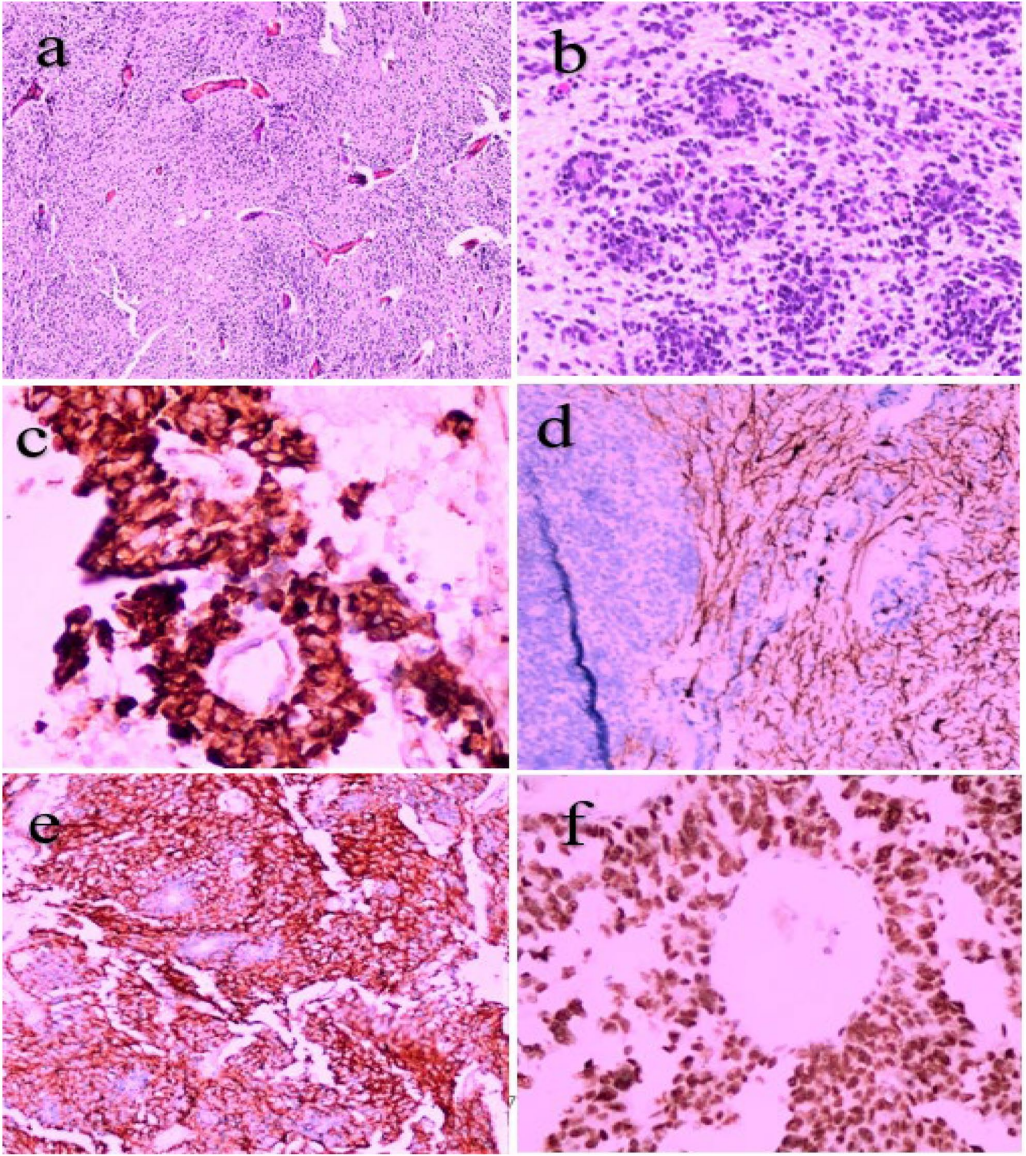
Histopathological and immunohistochemical features of an ETANTR (embryonal tumor with abundant neuropil and true rosettes) case. **a** Hematoxylin and eosin (H&E) stained section at 200× magnification illustrating a dense neuropil-rich background interspersed with true multilayered rosettes, characteristic of ETANTR morphology. **b** H&E-stained section at 400× magnification showing a well-formed rosette with clearly defined cellular architecture. **c** Immunohistochemical staining for LIN28A demonstrates strong cytoplasmic expression within the neuroepithelial tumor component, consistent with ETMR features. **d** GFAP highlights the neuropil-like fibrillary background, showing strong cytoplasmic staining, while rosettes remain negative. **e** Synaptophysin expression is diffuse within the neuropil-like matrix, with absence of staining in the rosettes, supporting neuronal differentiation. **f** Nuclear expression of INI-1 is retained, excluding atypical teratoid/rhabdoid tumor (AT/RT) from the differential diagnosis

**Fig. 3:**
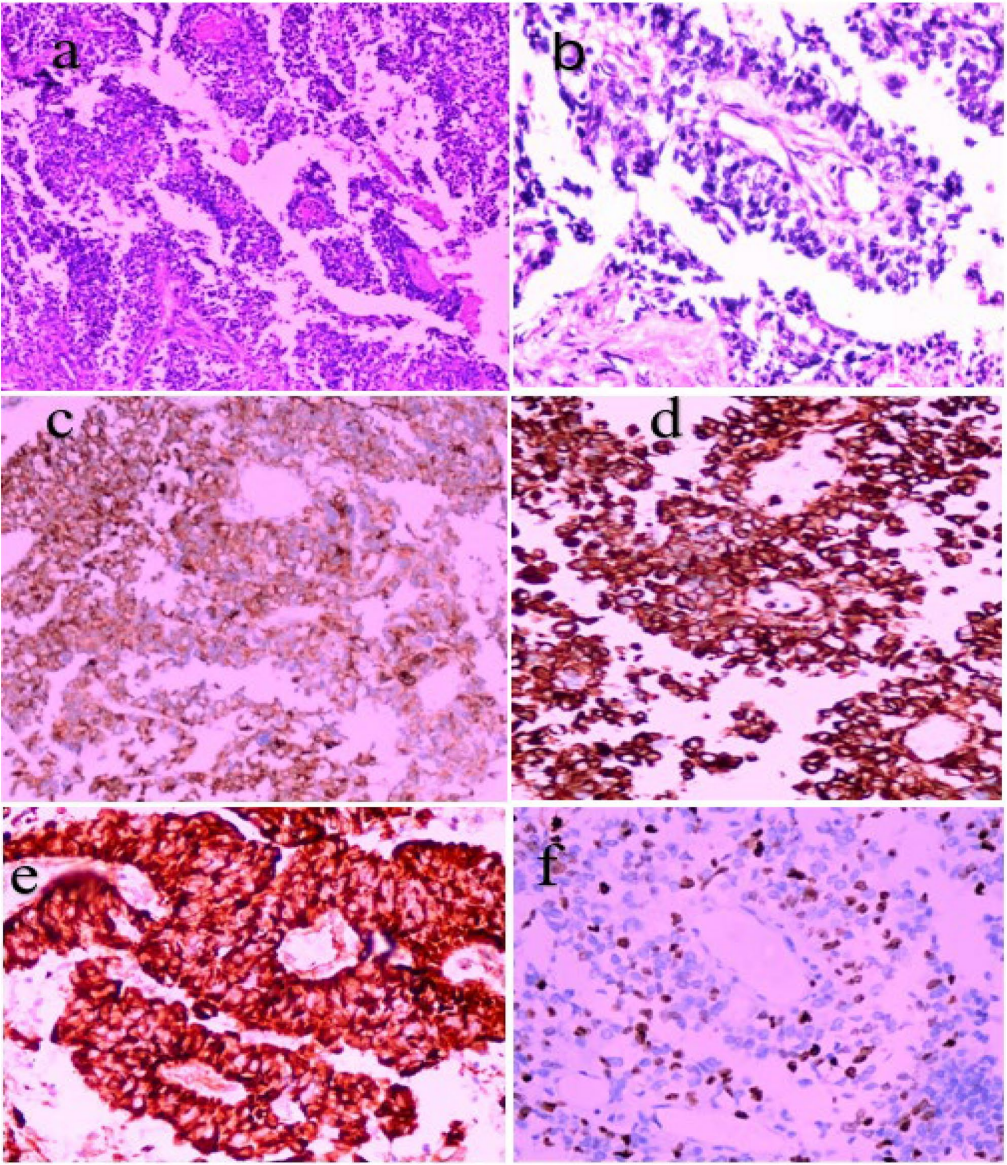
Histopathological and immunohistochemical features of a Medulloepithelioma case. **a** Hematoxylin and eosin (H&E) stain at 200× magnification revealing characteristic tubular formations and rosette-like structures. **b** Higher magnification view (H&E, 400×) highlighting the neuroepithelial architecture. **c** Immunohistochemistry demonstrating cytoplasmic positivity for LIN28A in neuroepithelial components. **d** Cytokeratin (CK) immunostain shows membranous and cytoplasmic expression in neuroepithelial cells. **e** Diffuse and strong positivity for Nestin, consistent with neural progenitor phenotype. **f** Ki-67 proliferation index estimated at approximately 30%, indicating a high proliferative activity

Figures 1 and 2, and 3 showcase the histopathological and immunohistochemical characteristics of representative cases of ependymoblastoma, ETANTR, and medulloepithelioma morphology, highlighting key diagnostic features and tumor-specific markers.

### Molecular analysis

Out of the 20 ETMR cases subjected to molecular analysis, C19MC amplification was detected in 18 cases, accounting for 90% of the cohort. Amplification was confirmed through quantitative PCR targeting representative loci within the C19MC cluster, with miR-517a and miR-520 g being the most frequently amplified, detected in 17/20 (85%) and 16/20 (80%) of analyzed cases, respectively. The remaining two cases (10%) lacked evidence of C19MC amplification. To further characterize these C19MC-negative tumors, targeted sequencing of the *DICER1* gene was performed. This revealed a pathogenic mutation in one case (5%), involving a known hotspot region associated with oncogenic activity in pediatric brain tumors, while the other case showed no detectable mutations in the regions examined. These results are consistent with existing literature, which reports that a small proportion of ETMR cases without C19MC amplification, typically ranging between 5% and 10%, may harbor somatic or germline *DICER1* mutations.

### Demographic characteristics and histological patterncorrelation

Among demographic parameters, patient age exhibited a statistically significant association with histological subtype (Monte Carlo *p* = 0.031; Cramér’s V = 0.562), indicating a moderate-to-strong relationship. For instance, younger children (particularly ≤ 4 years) more frequently exhibited features reminiscent of ETANTR, including abundant neuropil and multilayered rosettes, whereas older patients more frequently displayed patterns of ependymoblastic or medulloepithelial differentiation.Neither sex (*p* = 0.161) nor family history of malignancy (*p* = 0.136) showed significant correlation with histological classification. Although the duration of symptoms prior to diagnosis demonstrated a relatively strong effect size (Cramér’s V = 0.645), statistical significance was not reached (*p* = 0.527).

These findings suggest that age may play a pivotal role in histologic subtype predilection, potentially reflecting underlying biological divergence.

### Radiological and clinical correlates

Significant correlations were observed between histological subtype and variables such as tumor laterality, anatomical location (supratentorial versus infratentorial compartments), and radiological site (i.e. specific brain regions, e.g. the frontal lobe, cerebellum) (*p* = 0.034, 0.003, and < 0.001, respectively).ETANTR was more frequently identified in right-sided lesions, whereas ependymoblastomawas more common on the left. Larger tumor sizes were more often associated with ETANTR diagnoses. Supratentorial tumors (*n* = 26; 74.3%) were predominantly classified as ETANTR (*n* = 13; 37.1%). To evaluate the relationship between clinicopathological characteristics and histological subtypes, a thorough analysis was conducted. Tables 3 and 4 show the outcomes of a Monte Carlo simulation using Cramér’s V for effect magnitude. A distinct topographic preference was evident among the histological subtypes. While both ependymoblastoma (*n* = 10; 28.6%) and medulloepithelioma (*n* = 12; 34.3%) were found in infratentorial (*n* = 2, *n* = 7, respectively) and supratentorial (*n* = 8, *n* = 5, respectively) regions, ETANTR (*n* = 13; 37.1%) cases were exclusively supratentorial. Medulloepithelioma demonstrated a relatively balanced distribution with a subtle preference for infratentorial localization. Although ependymoblastoma appeared in both compartments (*n* = 10), it was more frequently supratentorial (*n* = 8). Importantly, tumor size was strongly linked to histological classification (Cramér’s V = 0.697; *p* < 0.001).ETANTR cases generally presented with larger lesions, frequently ranging between 6 and 9 cm, with multiple tumors measuring 8 cm or greater. Ependymoblastomas also showed relatively large dimensions, including several cases reaching 10 cm, though their size distribution appeared slightly broader, ranging from 5 to 10 cm. In contrast, medulloepitheliomas tended to be smaller overall, typically measuring between 4 and 7 cm. The statistically significant (*p* < 0.001) association between the ETANTR subtype and larger tumor growth is substantially influenced by anatomical location. Since all of the ETANTR cases (13/13) in our cohort were fully supratentorial—a compartment that allows for higher tumor development—the observed increased size is most likely due to this location rather than an independent biological feature of the subtype. Parameters including metastatic status, recurrence, and death did not demonstrate significant correlations (*p* > 0.05), although modest effect sizes were observed.

**Table 3:** Association between clinicopathological variables and histologic patterns based on Monte Carlo simulation and Cramér’s V effect size estimation. Strength of association was categorized as weak (Cramér’s V < 0.3), moderate (0.3–0.5), strong (0.5–0.7), and very strong (> 0.7). Statistically significant *p*-values (*p* < 0.05) are highlighted

Variable	χ² (df)	Monte Carlo Sig.	Cramer’s V	Association Strength
Laterality	13.373 (6)	0.034	0.437	Moderate
Topography	12.347 (3)	0.003	0.594	Moderate-Strong
Site of Tumor (Radiology)	85.548 (45)	< **0.001**	0.903	Very Strong
Size (cm)	50.938 (24)	< **0.001**	0.697	Strong
Metastases	4.029 (3)	0.301	0.339	Weak
Relapse	13.028 (9)	0.112	0.352	Weak
Death	13.018 (9)	0.119	0.352	Weak
No. of Rosettes / HPF	51.864 (15)	< **0.001**	0.703	Strong
Degree of Differentiation	43.400 (6)	< **0.001**	0.787	Strong
Atypia	25.975 (6)	< **0.001**	0.609	Moderate-Strong
Mitoses / 10 HPF	67.388 (18)	< **0.001**	0.801	Strong
Pattern of Necrosis	5.577 (3)	0.172	0.399	Weak
% Necrosis	46.350 (27)	0.048	0.664	Strong
Ki-67%	56.854 (27)	0.005	0.736	Strong
MVP (Microvascular Proliferation)	32.735 (6)	< **0.001**	0.684	Strong

Bold values indicate significance

**Table 4:** Association between selected clinicopathological parameters and histologic patterns in ETMR, assessed by Monte Carlo simulation with 10,000 iterations and Cramér’s V to quantify association strength. Interpretation of Cramér’s V: weak (< 0.3), moderate (0.3–0.5), strong (0.5–0.7), very strong (> 0.7). Statistically significant results (*p* < 0.05) are indicated

Variable	χ² (df)	Monte Carlo *p*	Cramér’s V	Association Strength
Laterality	13.373 (6)	**0.034**	0.437	Moderate
Topography	12.347 (3)	**0.003**	0.594	Moderate–Strong
Tumor Site (Radiology)	85.548 (45)	< **0.001**	0.903	Very Strong
Tumor Size	50.938 (24)	< **0.001**	0.697	Strong
Metastases	4.029 (3)	0.301	0.339	Weak

Bold values indicate significance

Collectively, these results underscore the value of imaging characteristics that exhibited correlations with specific histologic subtypes; however, accurate prediction of these subtypes requires integrating clinical factors.

### Histopathological parameters and their predictive value

Among the evaluated ETMR subtypes, significant histopathological differences were evident, with several features showing strong statistical associations with tumor classification. ETANTR cases consistently exhibited a higher number of multilayered rosettes per high-power field (typically 4–5), well-differentiated morphology, mild cytological atypia, and lower mitotic activity. These tumors also showed elevated proliferative indices, with Ki-67 levels frequently exceeding 60%, and were commonly associated with diffuse and extensive necrosis. In contrast, medulloepitheliomas demonstrated fewer rosettes, predominantly poor to moderate differentiation, marked atypia, variable mitotic counts, and Ki-67 indices ranging from 25% to 80%, with necrosis mainly focal. Ependymoblastomas presented intermediate features, typically with moderate differentiation and atypia, intermediate mitotic activity, variable necrosis patterns, and Ki-67 values often reaching up to 80%. Statistically, features such as rosette count, degree of differentiation, cellular atypia, and mitotic activity showed highly significant associations with histological subtype (all *p* < 0.001), with Cramér’s V indicating strong effect sizes. Additional parameters including necrosis percentage and Ki-67 index were also significantly correlated (*p* = 0.048), while necrosis pattern did not reach statistical significance (*p* = 0.172). Due to the limited sample size and categorical variables with low expected frequencies, Monte Carlo simulation was utilized to ensure robust assessment of associations. These findings underscore the diagnostic and prognostic value of both quantitative and semi-quantitative histological parameters in distinguishing ETMR subtypes, as detailed in Tables 3 and 4.

### Treatment response patterns across ETMR subtypes

**Fig. 4:**
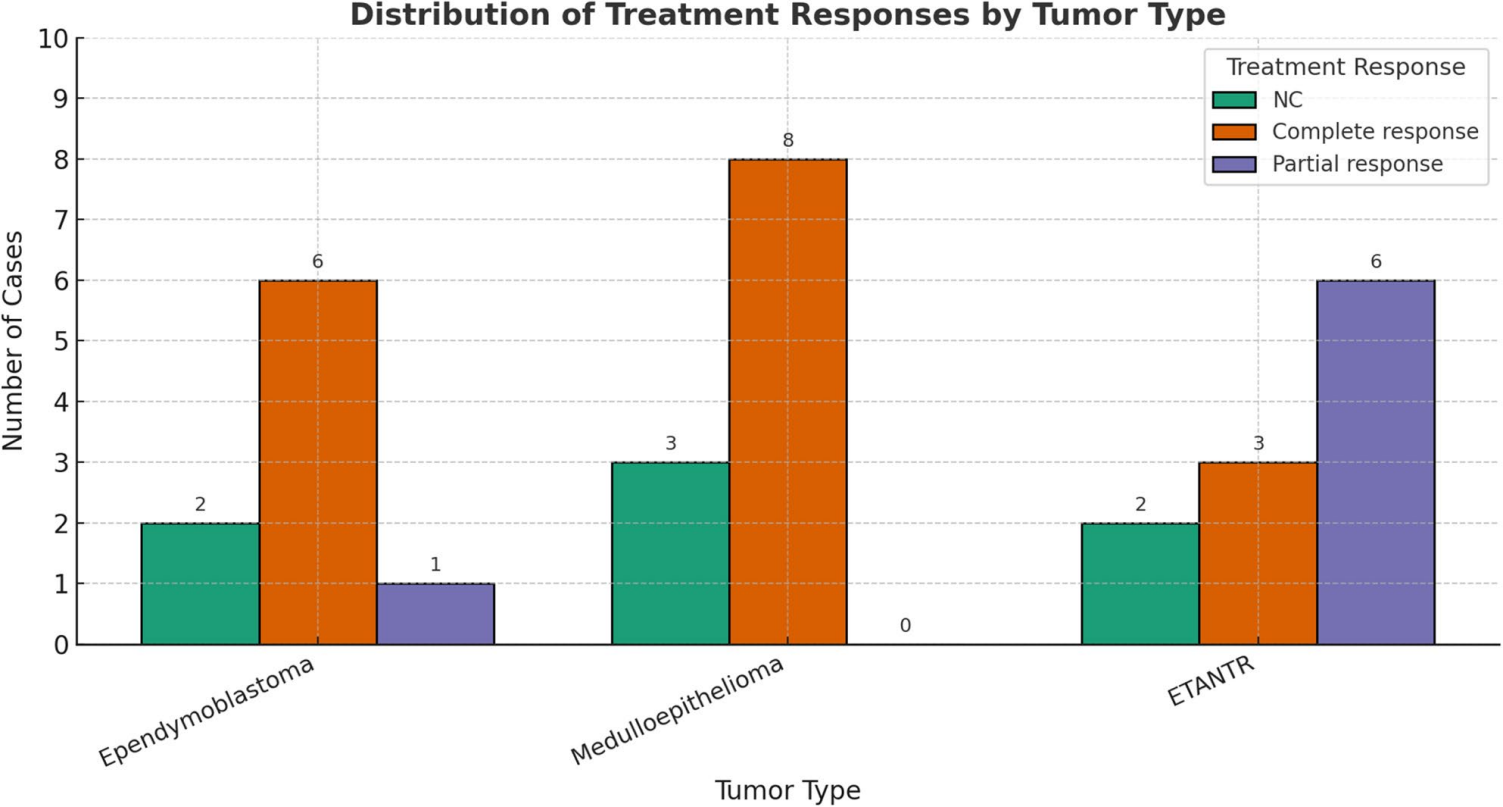
Analysis of treatment outcomes across ETMR subtypes revealed distinct response patterns. Medulloepitheliomas exhibited the highest frequency of complete responses, comprising eight of the total cases, followed by ependymoblastomas with six complete responses, and ETANTRs with three. ETANTR cases, however, showed the greatest proportion of partial responses (six cases), contrasting with only one such case among ependymoblastomas and none in medulloepitheliomas. NC indicates cases did not receive chemotherapy.These findings suggest subtype-specific variability in therapeutic responsiveness, with medulloepitheliomas being the most treatment-sensitive, while ETANTRs demonstrated a tendency toward partial response or resistance

In our cohort, all patients underwent a standardized therapeutic approach that included surgical resection, intensive chemotherapy, and radiotherapy. Analysis of treatment response across ETMR subtypes revealed notable variations in therapeutic outcomes. A complete response was most frequently observed in patients with medulloepithelioma (*n* = 9; 75%) and ependymoblastoma (*n* = 7; 70%), with several ETANTR cases (*n* = 4; 30.77%) also achieving complete remission. Partial responses were predominantly reported among ETANTR cases (*n* = 6; 46.15%). These findings suggest a variable treatment sensitivity across histological patterns, with ETANTR showing a broader spectrum of outcomes-ranging from complete to partial responses -whereas medulloepithelioma and ependymoblastoma demonstrated a higher likelihood of achieving complete remission. This underscores the importance of histological classification in predicting therapeutic outcomes in embryonal brain tumors (Fig. 4).

### Survival outcomes and prognostic analysis

Follow-up period ranged from 1.3 to 97.4 months, with a median follow-up duration of 19.0 months for overall survival (OS) and 19.6 months for progression-free survival (PFS). By the end of the study, 22 patients (62.9%) had died. The median OS was 19.0 months (95% confidence interval [CI]: 12.7–not reached), and the OS rate at final follow-up was 32.6% (95% CI: 19.5%–54.4%), with a restricted mean survival of 39.5 ± 7.29 months. Similarly, the median PFS was 19.6 months (95% CI: 14.0–not reached), with a progression-free survival rate of 23.4% (95% CI: 5.49%–99.4%) and a restricted mean PFS of 51.4 ± 8.28 months.

A comprehensive analysis of survival identified a number of clinicopathological characteristics as important predictors. Significantly linked to worse outcomes in the univariable study (Table 6) were pronounced atypia (PFS/OS *p* < 0.01), more rosettes (PFS *p* < 0.01, OS *p* = 0.037), and poor differentiation (PFS *p* = 0.013).

Interestingly, the histologic subtype was a significant predictor of Progression-Free Survival in this analysis (*p* = 0.022), with the ETANTR subtype exhibiting the shortest median PFS (12.0 months) in contrast to ependymoblastoma (56.5 months) and medulloepithelioma (median not achieved).

When stratified by cytologic atypia, the prognosis was greatly impacted by cytologic atypia, with mild and marked atypia exhibiting worse survival rates than moderate atypia. (Table 6). While ETANTR morphology was linked to the poorest overall survival (*n* = 9/13; 70%) –versus (6/10; 60%) in ependymoblastoma and (*n* = 4/12; 33%) in medulloepithelioma–, analysis stratified by age – 23 of 35 patients were 3 years old or less at the time of diagnosis, 11 cases of them exhibited poor PFS whereas 12 cases exhibited poor OS – showed no significant independent impact on either OS (χ² = 0.022, *p* = 0.882) or PFS (χ² = 1.280, *p* = 0.258), indicating that the unfavorable prognosis is largely determined by histologic subtype rather than patient age.

**Fig. 5:**
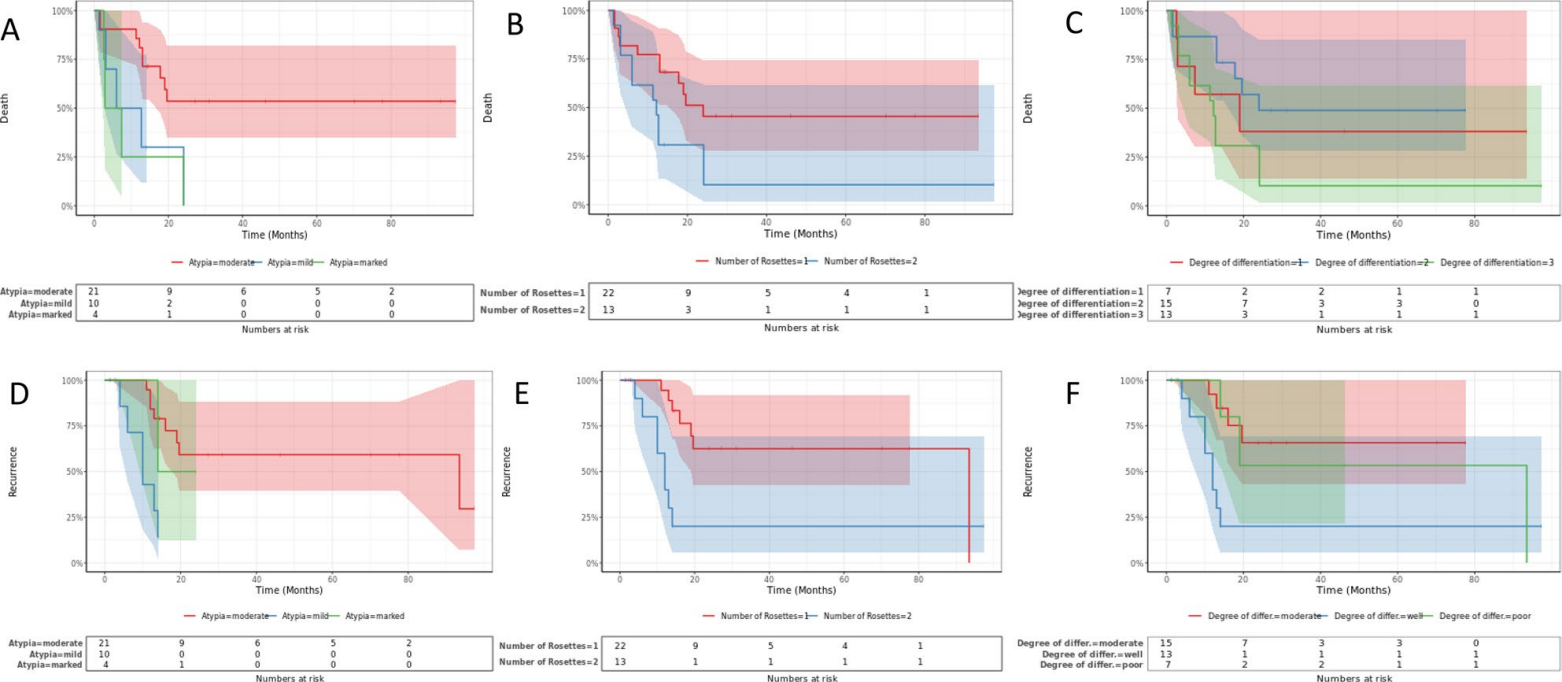
Impact of Histopathological Features on Survival and Recurrence in ETMR. Panel **A** (Death vs. Degree of Atypia): Overall survival is lowest in patients with significant atypia (median OS 5.15 mo). On the other hand, those with mild atypia have the best prognosis (median OS not achieved), with moderate atypia coming in second (median OS 9.35 mo). This difference was shown to be very significant (*p* < 0.01) using the log-rank test. Panel **B** (Death vs. Number of Rosettes): The group with two or more rosettes per high-power field shows a notably better survival trajectory compared to those with only one rosette. This trend implies that a higher number of rosettes might correlate with more differentiated tumor behavior and improved clinical outcomes. Panel **C** (Death vs. Degree of Differentiation): The most significant decrease in survival is seen in poorly differentiated cancers. Although this visual analysis does not show a clear prognostic differentiation between “Well” and “Moderate,” the survival outlook for well-differentiated and moderately-differentiated cancers is comparable and much better than for poorly-differentiated tumors. Panel **D** (Recurrence vs. Degree of Atypia): Marked atypia is associated with rapid and frequent recurrence, indicating a more aggressive disease course. Patients with mild atypia experience delayed and less frequent recurrences, supporting the notion that histological atypia is a strong predictor of recurrence risk. Panel **E** (Recurrence vs. Number of Rosettes): Tumors with ≥ 2 rosettes per field are less likely to recur, and recurrences, when present, tend to occur later. This contrasts with the group showing only one rosette, which demonstrates earlier and more frequent recurrences. This suggests that rosette count may reflect not only differentiation but also tumor aggressiveness. Panel **F** (Recurrence vs. Degree of Differentiation): The poorly differentiated group shows the highest and earliest recurrence rates. In contrast, well-differentiated tumors demonstrate relatively delayed and fewer recurrences. These patterns emphasize the predictive value of tumor differentiation for disease relapse

Notably, Kaplan-Meier and Cox regression analyses identified end-of-treatment evaluation as the most significant independent predictor for both OS and PFS (hazard ratio [HR] = 0.085; *p* < 0.001). Additionally, a higher Ki-67 proliferation index was associated with increased mortality risk (HR = 1.039 per 1%; *p* = 0.003), while histologic pattern significantly affected OS (HR = 2.677; *p* = 0.031). Interestingly, the absence of vomiting emerged as a strong protective factor for PFS (HR = 0.028; *p* = 0.002). Other independent prognostic factors for PFS included laterality, metastasis status, and degree of atypia, with severe atypia correlating with notably poorer survival outcomes. Collectively, these findings underscore the critical prognostic value of tumor biology, early treatment response, and disease characteristics in determining long-term survival trajectories in this high-risk pediatric population.Progression-free survival (PFS) and overall survival (OS) data for the study cohort are summarized in Table 5, while Table 6 details the univariable survival analysis assessing associations between clinicopathological variables and survival outcomes. Multivariable Cox regression analysis, presented in Table 7, identified key independent prognostic indicators for both PFS and OS. Notably, Fig. 5 illustrates that the severity of cytological atypia, the number of rosettes per high-power field, and the degree of histological differentiation significantly influence both survival and recurrence in ETMR, underscoring their prognostic importance.

**Table 5:** Descriptive summary of survival outcomes in 35 patients with ETMR. Data include median survival times with 95% confidence intervals (CI), number of events*, maximum follow-up duration, survival rates, and mean restricted survival times with standard errors (SE) for progression-free survival (PFS) and overall survival (OS)

Measure	PFS	OS
Median (95% CI)	19.6 (14.0; -)	19.0 (12.7; -)
Max Follow-up	97.4 months	97.4 months
Number of Patients (n)	35	35
Number of Events	15	22
Survival Rate (95% CI)	23.4% (5.49%; 99.4%)*	32.6% (19.5%; 54.4%)*
Mean Restricted Mean Survival (SE)	51.4 (± 8.28) months	39.5 (± 7.29) months

^*^* Disease progression, recurrence, or mortality are examples of PFS events. Death from any cause is considered an event for OS

The percentages indicate Kaplan–Meier–derived cumulative survival probabilities calculated at the most recent follow-up time point

**Table 6:** Univariable survival analysis correlating clinicopathological characteristics with progression-free survival (PFS) and overall survival (OS) outcomes in 35 patients with ETMR. The table presents median survival times in months with 95% confidence intervals (CI) for each category, along with corresponding p-values. Bold values highlight statistically significant findings (*p* < 0.05)

Variable	Category	PFS Median (months)	PFS *p*-value	OS Median (months)			OS *p*-value
Altered Consciousness	No	19.6 (14.0; -)	0.31	19.0 (12.7; -)		0.34	
	Yes	10.0 (10.0; -)		12.9 (1.6; -)			
Atypia	Mild	10.0 (6.0; -)	**< 0.01**	− (17.8; -)		**< 0.01**	
	Moderate	93.5 (19.0; -)		9.35 (3.10; -)			
	Marked	14.0 (14.0; -)		5.15 (2.60; -)			
Number of Rosettes	≤ 3	93.5 (19.6; -)	**< 0.01**	24.0 (17.8; -)		**0.037**	
	> 3	12.0 (10.0; -)		12.2 (6.0; -)			
Degree of Differentiation	Poorly differentiated	93.5 (19.0; -)	**0.013**	19.0 (2.90; -)		0.091	
	Moderately differentiated	− (19.6; -)		24.0 (17.8; -)			
	Well differentiated	12.0 (10.0; -)		12.2 (6.0; -)			
Evaluation at End of Treatment	Complete Response	93.5 (93.5; -)	**< 0.001**	- (-; -)		**< 0.001**	
	No Response	13.0 (11.0; -)		3.0 (1.6; -)			
	Partial Response	12.5 (12.0; -)		12.7 (12.2; -)			
Histologic Pattern	ETANTR	12.0 (10.0; -)	**0.022**	12.2 (6.0; -)		0.064	
	Medulloepithelioma	− (19.0; -)		− (19.0; -)			
	Ependymoblastoma	56.5 (16.0; -)		18.7 (13.0; -)			
Relapse	No	- (-; -)	**< 0.001**	- (-; -)		**< 0.001**	
	Yes	12.5 (11.0; 19.6)		13.0 (12.2; 24.1)			

Bold values indicate significance

**Table 7:** Results of the multivariable Cox proportional hazards regression analysis demonstrating independent prognostic variables associated with progression-free survival (PFS) and overall survival (OS) in patients diagnosed with ETMR. The table presents hazard ratios (HR) along with their 95% confidence intervals (CI) and corresponding p-values. Variables reaching statistical significance (*p* < 0.05) are indicated

Variable	Outcome	Hazard Ratio (95% CI)	*p*-value
**Evaluation at EOT**	OS &PFS	0.085 (0.029–0.253)	< 0.001
**Ki-67 (%)**	OS	1.039 (1.013–1.065)	0.003
**Histologic Pattern**	OS	2.677 (1.096–6.536)	0.031
**Vomiting**	PFS	0.028 (0.003–0.275)	0.002
**Laterality**	PFS	99.7 (5.8–1729.4)	0.002
**Metastasis**	PFS	27.2 (1.9–385.9)	0.015
**Atypia**	PFS	0.001 (0.000–0.045)	< 0.001
**Recurrence**	OS	3.48 (1.13–10.66)	0.03

Bold values indicate significance

Our findings affirm the highly aggressive nature of ETMRs. The majority of patients experienced recurrence and early mortality, despite multimodal treatment. Post-treatment response, Ki-67 index, and histologic subtype were the most robust predictors of OS. Interestingly, absence of vomiting and specific tumor lateralization were strong indicators of prolonged PFS.

These insights underscore the need for early detection, comprehensive histological assessment, and aggressive treatment approaches. Biomarkers like atypiaandKi-67 offer promising avenues for stratifying risk and tailoring therapy.

## Discussion

ETMR represent a highly malignant group of central nervous system neoplasms predominantly affecting infants and very young children. Historically classified into separate histological entities such as ETANTR, ependymoblastoma, and medulloepithelioma, these morphologically distinct tumors were unified in the 2016 World Health Organization (WHO) classification of CNS tumors based on their shared molecular signature—amplification of the C19MC locus on chromosome 19q13.42 [8, 9]. This molecular redefinition highlights the pivotal role of genomic profiling in refining tumor classification and guiding diagnostic criteria [21–24]. 

The current study substantiates previously documented epidemiological and clinical patterns associated with ETMRs. The median diagnostic age approximating three years and balanced gender distribution are congruent with existing literature, affirming the tumor’s predilection for early childhood [1, 3, 7]. Similar to earlier reports, the majority of patients presented with clinical manifestations attributable to increased intracranial pressure and focal neurological deficits, reflecting the mass effect and rapid growth kinetics characteristic of these tumors [2, 13]. Notably, a positive family history of malignancy remained an infrequent finding within this cohort, underscoring the largely sporadic nature of ETMR pathogenesis.

Anatomically, the cerebral hemispheres emerged as the predominant site of tumor origin, accounting for over 70% of cases, which is in line with the well-documented supratentorial predilection of ETMRs [1, 7, 9]. The typically large tumor volumes at diagnosis, coupled with radiological features of mass effect and midline shift, further illustrate the aggressive clinical trajectory of these lesions. Histopathological examination uniformly demonstrated a high proliferative index, with Ki-67 values averaging around 50%, alongside marked mitotic activity and extensive necrosis - parameters recognized as hallmarks of biologically aggressive ETMR [1, 2, 9].

Within this cohort, the distribution of ETMR histological subtypes was relatively balanced. However, statistical analyses revealed a significant correlation between patient age and tumor subtype, suggesting potential ontogenetic influences on tumor differentiation pathways. This observation aligns with prior comparative analyses proposing that ETMR phenotypic heterogeneity may, in part, reflect age-related biological distinctions [9, 10]. Furthermore, significant associations between histological subtypes and key pathological variables - including tumor size, rosette density, mitotic activity, and proliferative indices - imply that, despite their unified molecular background, distinct phenotypic patterns could influence clinical outcomes and therapeutic responsiveness [7, 9, 11].

From a neuroradiological perspective, this study reinforces the diagnostic relevance of anatomical localization and laterality, both of which demonstrated significant associations with histological subtypes. These findings support the established role of MRI-based evaluation, including tumor topography (spatial orientation within the brain; e.g., midline or lateralized lesions), in the early differential diagnosis of pediatric ETMR [10, 13]. Additionally, the observed concordance between radiological findings and pathological features highlights the indispensable value of integrated radiological–histopathological assessment in the diagnostic work-up of ETMR.

Prognostically, survival outcomes in this series remained dismal, with median overall and progression-free survival intervals of approximately 19 months, consistent with historical data underscoring the aggressive clinical course of ETMR despite multimodal therapy [11, 13]. Importantly, the study identified several independent prognostic indicators, including histological subtype, degree of cellular atypia, Ki-67 index, and radiological response at the end of initial therapy. Our multivariable Cox regression analysis, shown in Table 7, fully supports this conclusion. These parameters collectively emphasize the prognostic utility of both baseline tumor characteristics and early treatment response in informing patient outcomes and guiding clinical management strategies [11, 25].

The patients in this cohort received a variety of therapeutic treatments, most of which involved surgical resection. A subset of patients received adjuvant chemotherapy and/or radiotherapy (48% undergoing chemoradiotherapy). variability in response was influenced by patient- and disease-specific factors, particularly age and tumor size, with ETANTR morphology being more prevalent in younger patients presenting with larger tumors. Analysis of survival outcomes revealed that cytologic atypia significantly impacted prognosis, with Mild and Marked atypia showing reduced survival compared to tumors exhibiting moderate atypia. Although ETANTR morphology was associated with the poorest overall survival, age did not independently influence either OS or PFS, suggesting that long-term outcomes are predominantly determined by histologic subtype rather than patient age.

An intriguing observation in this study was the association between the absence of vomiting at presentation and prolonged progression-free survival. While the biological basis of this correlation remains uncertain, it may reflect the influence of tumor location on symptom development and subsequent timing of medical intervention, warranting further investigation in larger prospective cohorts.

Consistent with its recognized diagnostic role, LIN28A immunoexpression was diffusely and strongly positive in all cases examined. This finding corroborates earlier reports advocating LIN28A as a highly sensitive, though not entirely specific, immunohistochemical surrogate for identifying ETMR, particularly in resource-limited settings where comprehensive molecular testing may not be readily accessible [7, 10]. Nevertheless, reliance on LIN28A alone may be insufficient given its expression in other embryonal and high-grade pediatric CNS tumors, thus reinforcing the importance of integrating molecular analysis for C19MC amplification within diagnostic algorithms to ensure accurate tumor classification [8, 11].

While most ETMRs are defined by either C19MC amplification or DICER1 mutation, a minority of cases lacking both alterations have been increasingly recognized. In such instances, diagnosis depends on classical histological features - most notably, the presence of multilayered rosettes - and diffuse LIN28A overexpression. Recent literature estimates the frequency of genetically unclassified ETMRs at 1–5%, with possible etiologies including undetected genetic alterations, rare alternative oncogenic drivers, or technical limitations inherent to degraded archival tissue [9, 19, 20]. Korshunov et al. first detailed the existence of C19MC-negative, *DICER1*-wildtype ETMR-like tumors, highlighting the diagnostic value of morphology and LIN28A immunoreactivity in these diagnostically challenging cases [19]. This concept was validated, demonstrating that, although rare, such tumors share phenotypic and clinical parallels with genetically defined ETMRs and should prompt expanded molecular profiling, such as next-generation sequencing, to uncover potential alternative pathogenic events [20, 26].

In summary, this study reinforces the conceptual framework that ETANTR, ependymoblastoma, and medulloepithelioma constitute morphologic variants of a singular biological subtype – ETMR - characterized predominantly by C19MC amplification and universally aggressive clinical behavior [8, 9]. The robust correlations observed between histopathological features, imaging findings, and clinical outcomes underscore the necessity of comprehensive histopathological evaluation for risk stratification and prognostication. Furthermore, the prognostic significance of early treatment response highlights the urgency for prompt, aggressive, and multimodal therapeutic interventions.

These findings echo conclusions drawn from recent large-scale molecular analyses, which emphasize the centrality of genomic and epigenetic aberrations in ETMR pathogenesis, with potential implications for future targeted therapeutic strategies [11, 12].

This study offers important clinicopathological and prognostic observations in ETMR; however, certain methodological aspects warrant consideration. The retrospective nature of the work limits complete control over all clinical variables, though the use of standardized data collection methods and a unified therapeutic protocol helped mitigate heterogeneity. Subtle differences in patient-specific characteristics—such as age and tumor size—could not be fully eliminated but were systematically evaluated to reduce potential bias. The modest sample size reflects the exceptional rarity of ETMR, aligning with case numbers reported in other series, yet still enabled meaningful statistical analyses. While comprehensive molecular profiling was achieved in most cases, a small proportion lacked full testing due to limited tissue availability. For example, conventional histology and strong LIN28A positive were used to diagnosis a patient that tested negative for both C19MC amplification and DICER1 mutation. This could indicate an uncommon “driver-negative” ETMR, as reported in recent literature [27], or it could be the result of technical assay constraints with archival FFPE tissue. Nevertheless, the combined use of histopathology, immunohistochemistry, and available molecular data ensured a reliable diagnostic approach.

Moving forward, research efforts should prioritize the development of refined risk stratification tools, exploration of novel therapeutic agents, and elucidation of the biological mechanisms underlying ETMR heterogeneity and treatment resistance.

## Conclusion

ETMRs are rare, highly aggressive pediatric central nervous system tumors with a poor prognosis despite multimodal therapy. This study highlights the reproducible histomorphologic and immunophenotypic features of ETMR, particularly the diagnostic significance of multilayered rosettes and diffuse LIN28A expression, which remain valuable even in settings with limited resources. Adverse prognostic factors, including high mitotic activity, necrosis, and incomplete resection, underscore the tumors’ intrinsic aggressiveness and the critical role of gross total resection combined with adjuvant therapy. In the absence of widespread molecular testing, the integration of conventional histopathology with targeted immunohistochemistry provides a reliable diagnostic strategy. Identification of “ultra-high-risk” patients may be aided by knowledge of prognostic indicators including a high Ki-67 score and noticeable atypia. These results could be utilized to stratify individuals for inclusion in upcoming clinical trials or for evaluation of new therapeutic approaches, even though they are insufficient to de-escalate therapy for any subgroup. Ongoing multicenter collaboration and the development of innovative therapies are essential to improve survival for children with this devastating disease.

## Data Availability

All data underpinning the results of this study can be made available upon reasonable request to the corresponding author, in accordance with institutional and ethical guidelines.
